# Exploring the Genotype–Phenotype Correlation in *GBA*-Parkinson Disease: Clinical Aspects, Biomarkers, and Potential Modifiers

**DOI:** 10.3389/fneur.2021.694764

**Published:** 2021-06-24

**Authors:** Elisa Menozzi, Anthony H. V. Schapira

**Affiliations:** Department of Clinical and Movement Neurosciences, UCL Queen Square Institute of Neurology, London, United Kingdom

**Keywords:** Parkinson's, *GBA*, glucocerebrosidase, genotype-phenotype, biomarker

## Abstract

Variants in the glucocerebrosidase (*GBA*) gene are the most common genetic risk factor for Parkinson disease (PD). These include pathogenic variants causing Gaucher disease (GD) (divided into “severe,” “mild,” or “complex”—resulting from recombinant alleles—based on the phenotypic effects in GD) and “risk” variants, which are not associated with GD but nevertheless confer increased risk of PD. As a group, *GBA*-PD patients have more severe motor and nonmotor symptoms, faster disease progression, and reduced survival compared with noncarriers. However, different *GBA* variants impact variably on clinical phenotype. In the heterozygous state, “complex” and “severe” variants are associated with a more aggressive and rapidly progressive disease. Conversely, “mild” and “risk” variants portend a more benign course. Homozygous or compound heterozygous carriers usually display severe phenotypes, akin to heterozygous “complex” or “severe” variants carriers. This article reviews genotype–phenotype correlations in *GBA*-PD, focusing on clinical and nonclinical aspects (neuroimaging and biochemical markers), and explores other disease modifiers that deserve consideration in the characterization of these patients.

## Introduction

Biallelic pathogenic variants in the glucocerebrosidase (*GBA*) gene (OMIM 606463) leading to deficient activity of the lysosomal enzyme gene product GCase (EC 3.2.1.45) cause the recessively inherited multisystem disorder Gaucher disease (GD) (1). The standard classification of *GBA* variants is based on their phenotypic effect in GD, with “complex” rearrangements and “severe” variants causing neuronopathic GD, and “mild” variants causing non-neuronopathic GD (2). Patients with non-neuronopathic GD but especially heterozygous carriers of pathogenic *GBA* variants have an increased risk of developing Parkinson disease (PD) (3). Increased risk for PD has also been associated with a number of variants (p.E326K, p.T369M) not clearly pathogenic for GD (4, 5). In parallel with this increasing genotypic heterogeneity, detailed assessments of several cohorts of *GBA*-PD have suggested phenotypic differences associated with distinct genotypes.

Herein, we examine the clinical syndrome of *GBA*-PD compared to *GBA*-negative (noncarriers) PD and critically discuss genotype–phenotype correlation within the *GBA*-PD population from both clinical and biomarker perspectives. Finally, we present other potential disease modifiers that may impact on clinical phenotypes of *GBA*-PD.

## Methods

We performed a literature search for English-written publications on PD patients with *GBA* mutations using the National Center for Biotechnology Information's PubMed database (https://www.ncbi.nlm.nih.gov/pubmed) using the following search terms: “parkinson” AND “*GBA* OR 1q22” in the title and “gene OR genetic OR mutation OR mutated.” We selected the articles relevant to our review and included additional articles from their reference lists. *GBA* variants were defined as follows:

- “mild” and “severe” variants are determined as per the GD classification of variant severity
- “risk variants” are those that increase PD risk but are not pathogenic for GD
- “complex” variants result from conversions, fusions, and insertions of parts of the pseudogene *GBAP1* into *GBA*.

## Genotype–Phenotype Correlation With Clinical Profile in *GBA*-Parkinson Disease

### Epidemiological, Demographic, and Prognostic Features

The frequency of *GBA* carriers is population-specific and ranges from 10% to 31% in Ashkenazi Jewish (AJ) to 3% to 12% in non-AJ North American (with European background) populations (6). As an example of ethnic heterogeneity of *GBA* variants, the p.N370S variant is very common in AJ populations with European origin and non-AJ European/West Asian populations, whereas it is not generally detected in East Asian populations (7–9). The penetrance of *GBA* variants in PD is low, age-specific, and controversial across studies, with an estimated 9.1% of carriers developing PD over their lifetime (10). At age 60 and 80 years, PD risks of ~5 and 9%−12% respectively are reported among GD patients, which is significantly greater than those of noncarriers (0.7 and 2.1%, respectively) but similar to the 1.5–14% and 8–19% respective prevalence among *GBA* heterozygote carriers (11–15), suggesting that PD risk is not further increased by carrying a second *GBA* mutant allele (11). Among familial PD cohorts, much higher penetrance is reported (13.7% at 60 years and 29.7% at 80 years) (16), though this is likely a contribution from other genetic factors in these cohorts. When considering the impact of different variants on penetrance, the odds ratio (OR) of developing PD was found to be much higher in people carrying severe rather than mild variants (10.3–13.6 vs. 2.2) (17, 18). However, no such differences in penetrance were reported between mild and severe variants in familial PD cases (16), again suggesting the influence of additional genetic factors in such cohorts. The most frequent risk variant associated with *GBA*-PD, p.E326K, has been associated with a PD OR of 1.60–2 in European PD populations and up to 5.5 in AJ patients (19–21), suggesting a similar if not higher risk compared with mild variants.

Compared with noncarriers, *GBA*-PD patients usually present symptoms earlier on (6, 22–26). PD patients with biallelic *GBA* variants (either homozygous or compound heterozygous), hereafter referred to as GD-PD, also have an earlier age at onset compared with heterozygous carriers (11, 27), indicating a possible “dose” effect of *GBA* influencing age at onset. When *GBA* variants were stratified, the majority of studies consistently reported earlier age at onset in severe variant carriers compared with mild or risk (17, 22, 28) and in patients with null or complex alleles relative to those with missense mutations (29). Rarely, no differences in age at onset were found (30, 31).

In terms of disease progression, carriers of any *GBA* variant reach progression milestones earlier compared with noncarriers (23, 32). In one study evaluating the impact of different variants on survival, severe and mild variants (considered together) were associated with a 2-fold greater risk of death with mean time to mortality approximately 1 year earlier compared with noncarriers, whereas risk variants showed similar mortality rates and time to mortality compared with noncarriers (32). In another longitudinal study conducted on PD patients of AJ ancestry, though, no significant effect of either mild or severe variants on survival was found (33). When survival of patients with mild and severe variants was directly compared, no differences were reported; nevertheless, only severe variants were associated with a greater risk of death relative to noncarriers (34). Overall, these results could suggest that only severe mutations might confer poor survival rates.

### Motor and Nonmotor Features

Compared with noncarriers, the usual presentation of *GBA*-PD is that of an akinetic-rigid syndrome, with early development of motor fluctuations and dyskinesia (22, 29, 35). Stratifying by *GBA* variants, severe variants have been associated with more severe and rapidly progressive motor phenotype, and shorter time to development of axial symptoms such as postural instability, as opposed to mild or risk variants (22, 32). At the other end of the spectrum, risk variants are more likely to associate with benign phenotypes and occurrence of motor fluctuations later in the disease course (22).

People with *GBA*-PD suffer from a higher burden of nonmotor symptoms both in the prodromal phase and during manifest disease. One study reported higher scores of the nonmotor symptoms scale in *GBA*-PD patients compared with noncarrier PD patients (12). Similar results were confirmed by another study showing that PD patients with severe variants or GD-PD patients had higher nonmotor symptoms questionnaire scores compared with PD patients carrying mild variants or noncarriers (31).

Among the nonmotor features associated with PD, hyposmia, constipation, and REM sleep behavior disorder (RBD) are the most important prodromal risk factors for PD, and hereafter, we will discuss them individually. Olfactory function has been consistently shown to be worse and deteriorate over time in asymptomatic *GBA* carriers and GD patients compared with noncarriers (36–38). Interestingly, one study reported that more severe hyposmia at baseline could predict the development of parkinsonism in these individuals (39). Poorer hyposmia has also been reported in PD patients carrying pathogenic (severe/mild) variants vs. noncarriers (40), as well as in asymptomatic carriers of severe vs. mild variants (31). Moreover, GD-PD patients showed more severe loss of olfaction compared with *GBA* heterozygous and noncarrier PD patients (27), suggesting that possibly not only type but also “dose” of *GBA* variants may affect olfactory function. Regarding constipation, only few reports have investigated its occurrence separately from other autonomic features in *GBA*-PD, finding that constipation may present more frequently relative to noncarriers (41, 42). Data regarding RBD are controversial. On the one hand, RBD has been reported to occur more frequently in *GBA*-PD and GD-PD compared with noncarrier PD patients (28, 42) and in PD patients carrying severe variants compared with patients carrying mild variants (31). On the other hand, no differences were reported in cohorts of *GBA* carriers or GD patients in comparison to noncarrier healthy controls (36). In a longitudinal evaluation study, *GBA* carriers and GD patients showed a worsening of RBD symptoms over time (39); however, whether a deterioration of RBD symptoms in these subjects leads to the development of PD is not known.

After the diagnosis of PD, *GBA*-PD patients show an increased risk of cognitive decline (22, 23, 25, 26, 41, 43, 44), including those receiving deep brain stimulation surgery (45, 46). Specific cognitive domains seem to be more affected, particularly in visual short-term memory (47). When stratified by variant, in one study, PD carriers of severe or biallelic variants showed worse cognitive function compared with noncarriers, whereas mild or risk variants did not (30). However, other studies found that risk variants (p.E326K) were associated with similar cognitive deterioration (26, 48), or faster progression to dementia compared with pathogenic *GBA* variants (49, 50), as opposed to what is expected on the basis of the impact on GCase activity.

Increased frequency of psychiatric symptoms, such as hallucinations, delusions, and impulsive–compulsive behavior (ICB), has also been reported in *GBA*-PD patients vs. noncarriers (22, 41, 44, 51). The risk of psychiatric disturbances seems to be genotype specific. Severe or complex variant carriers were more affected than mild variant carriers (22, 31), and risk variant carriers showed the mildest phenotype (22).

Regarding autonomic function, this has been reported to be more affected in *GBA*-PD (22, 41, 42, 44), but no association of type or “dose” of *GBA* variants with autonomic phenotypes has been reported to date (22, 27).

In summary, within *GBA*-PD patients, motor symptoms, psychiatric disturbances, and possibly hyposmia are more severe and might show genotype–phenotype correlations. The genotype–phenotype association for cognitive and autonomic function is less clear, although these features are clearly more severely affected. Whether constipation and RBD are overrepresented features in *GBA*-PD or asymptomatic *GBA* carriers, and a genotype–phenotype correlation exists for these symptoms, has not yet been elucidated. The more rapid decline in motor and nonmotor features in *GBA*-PD and the influence of specific *GBA* variants in these patients should be considered in the context of personalized treatment strategies. For instance, clinicians should be particularly cautious in the use of medications increasing the risk of falls, or worsening autonomic function in *GBA*-PD patients, and should recommend to these patients to start physiotherapy and cognitive engagement strategies early in the disease course (52).

## Genotype–Phenotype Correlation With Nonclinical Biomarkers in *GBA*-Parkinson Disease

### Neuroimaging

#### Presynaptic Dopamine Terminal Function

The degree of dopaminergic dysfunction in *GBA*-PD has been evaluated in few studies. Cilia et al. (34) showed that compared with noncarriers, PD patients carrying a severe (but not mild) variant had a significant dopamine transporter (DAT) deficit. When mild and severe variant carriers were directly compared, individuals with severe variants showed more pronounced deficit, mainly in the striatum contralateral to the most affected side (34). One study evaluating a small cohort of PD patients carrying risk variants (p.E326K and p.T369M) vs. noncarriers reported a reduced [^18^F]FDopa uptake in the bilateral caudate nuclei, anteromedial putamen ipsilateral, and nucleus accumbens contralateral to the most affected site in carriers (53), but no comparison was made with patients carrying other variants. Surprisingly, in a cohort of early PD patients mostly carrying mild *GBA* variants (89% p.N370S), patients showed higher specific binding ratio (SBR) in the contralateral caudate and putamen when compared with noncarriers (54), and higher SBR values in caudate, putamen, and striatum were also reported in non-manifesting p.N370S carriers relative to healthy controls (55). The proposed mechanisms underlying this observation might be either of a compensatory upregulation of tracer uptake in the early stage of the disease (associated with slower decline rate in DAT signal) or the result of disruption of dopamine release prior to dopaminergic terminal loss (54, 55). Longitudinal assessments evaluating DAT deficit progression in *GBA* carriers bearing different variants will elucidate the implicated mechanisms.

#### Brain Metabolism

Metabolic networks have been investigated in *GBA*-PD patients using [^18^F]-FDG PET, suggesting greater disease activity compared with noncarriers, as shown by increased PD-related pattern (PDRP) and a trend toward increased PD-related cognitive pattern (PDCP) levels (56). Recently, Greuel et al. (53) reported a similar pattern in a small cohort of PD patients carrying risk variants (p.E326K and p.T369M), with both higher PDRP and PDCP levels and significant [^18^F]-FDG PET hypoactivity in the parietal lobe. These findings reflect the higher cognitive burden seen in *GBA*-PD and suggest that even risk variants such as p.E326K and p.T369M might be associated with a severe cognitive decline.

#### Substantia Nigra Hyperechogenicity

In a cross-sectional study using transcranial sonography, both asymptomatic *GBA* heterozygous carriers and GD patients showed an enlarged hyperechogenic area of the substantia nigra compared with healthy controls, but longitudinal studies are needed to determine the predictive value of these findings (57). In manifest PD, transcranial sonography could not discriminate between *GBA*-PD and noncarriers, although a higher percentage of *GBA*-PD patients showed interrupted brain stem raphe, a marker of serotonergic system impairment (41).

#### Brain Atrophy

Segmentation of cortical and subcortical structures can provide information about regional atrophy. By comparing *GBA*-PD and *GBA* asymptomatic carriers vs. noncarrier PD and healthy controls, lower structural volumes and widespread cortical thinning were found among patients with PD compared with asymptomatic participants, but none of these differences were related to the genetic status (58). Given the more severe clinical profile associated with *GBA*-PD, one would have expected a more diffuse impairment in these individuals and possibly even in the asymptomatic carriers. Therefore, the applicability of this tool remains uncertain.

### Biochemical Markers

#### Alpha-Synuclein

Total α-synuclein has been evaluated in the cerebrospinal fluid (CSF) of *GBA*-PD patients, showing lower levels compared with noncarriers (59). After genotypic stratification, severe variants displayed the lowest levels, and mild variants had lower levels compared with risk variants, suggesting a genotype–phenotype association (28). Interestingly, a similar correlation between genotype and CSF α-synuclein has been found in cohorts of patients with Lewy body dementia carrying *GBA* variants (60).

Plasma oligomeric α-synuclein levels are considered one of the major factors in neurodegeneration in PD (61). *GBA*-PD patients showed increased levels compared with noncarriers, with a trend toward higher levels in those carrying severe/mild variants followed by risk variants (62). The possible association of plasma oligomeric α-synuclein and severity of *GBA* variants reinforces the hypothesis that decreased GCase enzymatic activity plays a central role in PD pathogenesis.

The presence of phospho-α-synuclein pathology in skin biopsies has been evaluated in one study of 10 *GBA*-PD patients (six p.N370S, three p.E326K, one p.L444P). Six out of 10 demonstrated phospho-α-synuclein deposition, mainly in autonomic but also somatosensory fibers (63). These findings resemble what is seen in PD noncarriers, suggesting that skin biopsies might be used to investigate α-synuclein pathology *in vivo*, but they might not be useful to discriminate among different *GBA* genotypes.

#### Metabolic Fingerprints: Glucocerebrosidase Activity, Lysosphingolipids, and Others

Enzymatic activity of GCase seems to be a promising biomarker in *GBA*-PD, showing a genotype–phenotype association. Lower GCase enzymatic activity measured in dried blood spots has been reported in *GBA*-PD patients compared with noncarriers (64), and after genotypic stratification for *GBA* variants, increasing severity was associated with decreasing residual GCase activity (22, 62, 65) and longitudinally with a steeper decline of enzymatic activity (65). When measured in CSF, GCase activity was again significantly reduced in *GBA*-PD (66). Investigating how single *GBA* variants affect CSF GCase levels and whether they correlate with levels measured in dried blood spots might be of particular interest.

Lipid dysregulation has been proposed as one of the pathogenic mechanisms underlying *GBA*-PD (67). Elevation of different lipids, such as ceramide, total monohexosylceramide (glucosylceramide + galactosylceramide), sphingomyelin, and sphingosine (glucosylsphingosine + galactosylsphingosine), has been reported in *GBA*-PD vs. noncarriers (68). Galactosylsphingosine and glucosylsphingosine tended to be higher in patients carrying severe/mild variants compared with risk variants (69); however, their elevation was not correlated with either GCase activity measured in dried blood spots or plasma α-synuclein levels (69), arguing against a causal relationship between GCase deficiency and substrate accumulation.

To date, one study has evaluated the metabolomic profile of *GBA*-PD patients using gas chromatography/mass spectrometry. Using untargeted approach, elevated levels of several amino acids including asparagine, ornithine, glutamine, glycine, and polyol pathway metabolites were found in plasma of *GBA*-PD patients carrying risk variants compared with noncarriers (53). Interestingly, the two groups were substantially identical in terms of clinical features, suggesting that assessing the metabolomic profile might be a good biomarker to differentiate patients in early stages.

#### Inflammatory Mediators

Biomarkers of systemic inflammation have been investigated in few studies in *GBA*-PD, with contrasting results. In one study, higher levels of interleukin-8 differentiated *GBA*-PD from noncarriers and were associated with poorer cognitive function (43). The same study also reported elevation of other cytokines, such as monocyte chemotactic protein-1 (MCP-1) and macrophage inflammatory protein-1α in *GBA*-PD, whereas another study reported reduced levels of MCP-1 (70). The discrepancy between these results might be due to small sample sizes, different methodologies, and perhaps lack of stratification by variant type.

Overall, multiple biomarkers have been proposed so far in *GBA*-PD. Dopaminergic imaging and metabolic imaging seem promising candidates to elucidate possible genotype–phenotype correlations. Reduced total CSF α-synuclein, increased plasma oligomeric α-synuclein, and reduced GCase activity have shown genotype–phenotype associations that would require further confirmation in future studies. Whether lipidic and metabolic profiles are influenced by genotype remains elusive. Furthermore, the application of validated biomarkers to the prodrome and progression of PD in general remains a controversial area (71).

The most important clinical and nonclinical data about genotype–phenotype correlations in *GBA*-PD are summarized in Table 1.

**Table 1:** Summary of main studies reporting genotype–phenotype correlations in *GBA*-Parkinson Disease.

**Study Ref**	**Patient Groups (n. of patients)**	**Clinical features**			**Nonclinical biomarkers**
		**AAO, mortality**	**Motor features**	**Nonmotor features**	
Lerche et al. (28)	M (16) vs. S (21) vs. R (43)	Younger AAO (S vs. R)	No difference	Highest history of dementia (S)	Low CSF total α-synuclein (S < M < R)
Huh et al. (65)	M (15) vs. S^*^ (6) vs. R (26)	NA	NA	NA	Reduced GCase activity (S < M < R)
Petrucci et al. (22)	M (32) vs. S (39) vs. R (24) vs. C (16)	Younger AAO (S vs. M+C+R)	Lower AKR onset (R vs. M+S+C)	Lower risk of ICB (R)Lower risk of dementia and delusions (M)	Reduced GCase activity (C < S < M < R)
Cilia et al. (34)	M (67) vs. S (56)	Trend toward younger AAO (S)Higher risk of death compared to noncarriers (S)	No difference	Higher risk of dementia (S)	Reduced parieto-occipital blood perfusion (S) More pronounced nigrostriatal terminal reduction (S)
Liu et al. (30)	M (28) vs. S (26) vs. R (127) vs. D (14)	No difference in AAO	No difference	Higher risk of cognitive decline (S, D)	NA
Stoker et al. (32)	M+S (17) vs. R (31)	Higher risk of mortality compared with noncarriers (M+S)	Increased risk of postural instability (M+S)	Higher risk of dementia (M+S)	NA
Malek et al. (40)	M+S (48) vs. R (117); early disease stage (duration from diagnosis less than 1.5 years)	Younger AAO compared with noncarriers (M+S)	More severe symptoms compared with noncarriers (M+S)	More frequent hyposmia compared with noncarriers (M+S)No difference in cognitive function compared with noncarriers (M+S, R)	NA
Pchelina et al. (69)	M+S (11) vs. R (12)	NA	NA	NA	Trend toward higher levels of hexosylsphingosines (M+S > R)
Pchelina et al. (62)	M+S (11) vs. R (11)	NA	NA	NA	Trend toward reduced GCase activity (M+S < R) Trend toward higher plasma oligomeric levels of α-synuclein (M+S > R)
Thaler et al. (31)	M (139) vs. S (48) vs. D (16)	No difference in AAO	More severe symptoms (S, D vs. M)	More severe hyposmia, nonmotor symptoms, depression (S)Higher frequency of RBD (S, D vs. M)	NA
Gan-Or et al. (18)	M+S (71) vs. D (6)	Younger AAO (D vs. M+S)	No difference	NA	NA

*AAO, age at onset; AKR, akinetic-rigid; C, complex variant (defined as two or more variants in cis as the result of conversion, fusion, insertion of parts of GBAP1 into GBA); CSF, cerebrospinal fluid; D, dual (more than one GBA variant); GBA, glucocerebrosidase gene; GCase, glucocerebrosidase enzyme; ICB, impulsive–compulsive behavior; M, mild variant; NA, not applicable; PD, Parkinson disease; R, risk variant; RBD, REM sleep behavior disorder; S, severe variant;*

* *, in this study, severe GBA-PD patients also include homozygous and compound heterozygous carriers of any GBA variant*.

## Potential Disease Modifiers in *GBA*-Parkinson Disease

Aside from direct genotype–phenotype correlations within *GBA*-PD, several other genetic and environmental factors may influence both disease penetrance and clinical features. These are important to consider and control for when evaluating *GBA*-PD cohorts to avoid erroneous causal attribution of observed symptoms to *GBA* genotype alone.

Among genetic factors, common single-nucleotide polymorphisms (SNPs) within the *GBA* locus have been proposed as potential modifiers of *GBA*-PD age at onset and motor progression (72, 73). Beyond *GBA*, the Alzheimer disease Bridging Integrator 1 (*BIN1*) locus (OMIM 601248), which is involved in synaptic vesicle endocytosis in the central nervous system, has also been proposed as a modifier of age at onset in *GBA*-PD, with the rs13403026 SNP being associated with older age at onset in both mild and severe *GBA* carriers (74). Another candidate is the Metaxin 1 (*MTX1*) gene (OMIM 600605), which is located close to *GBA* and encodes a mitochondrial protein. Homozygous c.184A/A genotype in the *MTX1* gene is associated with earlier age at onset in *GBA*-PD (75). Using a genome-wide association study, specific variants in close proximity to α-synuclein (*SNCA*; OMIM 163890) and cathepsin B (*CTSB*; OMIM 116810) genes (rs356219 and rs1293298) were found to associate with earlier age at onset in *GBA* carriers (76). Interestingly, the G/G *SNCA* rs356219 genotype was also associated with a more aggressive phenotype in a small cohort of *GBA*-PD patients (77). These findings suggest a possible synergistic effect of *GBA* and *SNCA* variants and deserve further evaluation in stratified groups of *GBA* carriers.

Although the mechanisms by which GCase influences PD pathogenesis are still debated (78), any factor influencing lysosomal GCase activity might potentially be disease modifying. For instance, it has been demonstrated that α-synuclein itself can induce aberrant maturation and endoplasmic reticulum/Golgi apparatus trafficking of GCase and therefore reduce the mature form of GCase and its lysosomal activity (79). More recently, it has been suggested that mutant leucine-rich repeat kinase (*LRRK2*; OMIM 609007) products may act as a negative regulator of GCase activity. GCase activity was shown to be reduced in human dopaminergic neurons carrying different *LRRK2* mutations, and the treatment of dopaminergic neurons from patients with either *LRRK2* or *GBA* variants with LRRK2 kinase inhibitor could increase GCase activity and rescue neurons from PD-related damage (80).

Among non-genetic risk factors, a recent study conducted on a large cohort of asymptomatic *GBA* carriers has evaluated the role of metabolic syndrome, a well-known risk factor for PD (81), as a possible disease determinant. The authors did not find any association between metabolic syndrome and risk of PD; however, hypertriglyceridemia and prediabetes were possibly overrepresented in those destined to later develop PD, regardless of *GBA* genotypes (82). In one study evaluating multiple environmental factors linked to PD, a more frequent exposure to pesticides was reported in *GBA*-PD patients vs. noncarriers, whereas no difference in smoking or coffee drinking was found (83). These preliminary data need further validation but may suggest that certain components of metabolic syndrome such as insulin resistance, or previous exposure to pesticides, should be carefully considered as potential disease determinants in *GBA* carriers.

## Conclusions

Increasing numbers of *GBA* variants have been associated with an elevated risk of PD, but the standard classification of *GBA* variants incompletely reflects the complex and rapidly evolving genetic landscape of *GBA*-PD. Moreover, data from cohorts of *GBA*-PD patients suggest that carriers of different variants display specific clinical profiles, with complex or severe variants associated with a more aggressive and rapidly progressive PD phenotype and mild or risk variants with a more benign phenotype.

Stratifying *GBA* carriers in both the prodromal and manifest phase of PD is of paramount importance, first, to address questions about prognosis, advanced treatment response, and counseling and, second, to recognize early the presence of subclinical/clinical symptoms that might help more precise selection of individuals for clinical trials. Multimodal evaluations including metabolic imaging and assessment of GCase activity, α-synuclein levels, and lipid and metabolic profile may shed light on inter-genotype differences, discover new biomarkers for clinical and research setting, and unveil novel mechanisms underlying *GBA*-PD pathogenesis.

## Author Contributions

EM conceived the manuscript and wrote the first draft. AS conceived and reviewed the manuscript. Both authors contributed to the article and approved the submitted version.

## Conflict of Interest

The authors declare that the research was conducted in the absence of any commercial or financial relationships that could be construed as a potential conflict of interest.
